# College Announcements

**Published:** 1874-08

**Authors:** 


COLLEGE ANNOUNCEMENTS.—Savannah Medical College.
Medical Department University of Georgia.
University of Nashville, Department of Medicine and Surgery.
Atlanta Medical College.
Charity Hospital, Medical College, New Orleans.
Medical College of Ohio.
Alabany Medical College.
College of Physicians and Sugeons, New York.
University of the city of New York.
Medical College of the State of South Carolina.
Texas Medical College and Hospital.

